# The analysis of clonal expansions in normal and autoimmune B cell repertoires

**DOI:** 10.1098/rstb.2014.0239

**Published:** 2015-09-05

**Authors:** Uri Hershberg, Eline T. Luning Prak

**Affiliations:** 1 School of Biomedical Engineering, Science and Health Systems, Drexel University, Bossone 7-711, 3141 Chestnut Street, Philadelphia, PA 19104, USA; 2 Department of Immunology and Microbiology, College of Medicine, Drexel University, Bossone 7-711, 3141 Chestnut Street, Philadelphia, PA 19104, USA; 3 Department of Pathology and Laboratory Medicine, Perelman School of Medicine, University of Pennsylvania, 405B Stellar Chance Labs, 422 Curie Boulevard, Philadelphia, PA 19104, USA

**Keywords:** antibody, clone, clonotype, immunoglobulin, high-throughput sequencing, repertoire

## Abstract

Clones are the fundamental building blocks of immune repertoires. The number of different clones relates to the diversity of the repertoire, whereas their size and sequence diversity are linked to selective pressures. Selective pressures act both between clones and within different sequence variants of a clone. Understanding how clonal selection shapes the immune repertoire is one of the most basic questions in all of immunology. But how are individual clones defined? Here we discuss different approaches for defining clones, starting with how antibodies are diversified during different stages of B cell development. Next, we discuss how clones are defined using different experimental methods. We focus on high-throughput sequencing datasets, and the computational challenges and opportunities that these data have for mining the antibody repertoire landscape. We discuss methods that visualize sequence variants within the same clone and allow us to consider collections of shared mutations to determine which sequences share a common ancestry. Finally, we comment on features of frequently encountered expanded B cell clones that may be of particular interest in the setting of autoimmunity and other chronic conditions.

## 1. Introduction

B cell clones comprise the fundamental units of selection in the humoral immune response. But what defines a clone? Clonally related cells derive from a common progenitor cell. All cells in the body initially derive from the zygote, but saying that all of the cells in the body are clonally related is not a satisfying answer for B cells. B cells undergo somatic recombination events and somatic hypermutation (SHM) to create a diverse repertoire of clones. To find a better answer to this question, we need to think of differentiation events and how they limit downstream cell fates. Therefore, in §2, we begin by reviewing basic checkpoints in B cell development where the antibody repertoire is generated, diversified and selected. Clones, or collections of genetically similar cells (as defined by similarities in their antibody heavy chain, light chain or a combination of both), emerge at these different checkpoints. How clones are defined is also profoundly influenced by the kind of experiment that is performed. In §3, we review four basic kinds of repertoire experiments and comment on the advantages and disadvantages of each with respect to clone identification and characterization.

Next, we come to the computational problem of identification and analysis of clones in high-throughput sequencing data. In §4, we describe different methods for defining clones and some of the errors that arise with these different approaches. We emphasize the need for trying different methods and using different parameters for defining clones to determine if clonal lineages are robust. In §5, we comment on clonal expansion, diversity and overlap. Here, we focus primarily on the diversity of the repertoire as a whole (inter-clonal diversity). In §6, we comment on two general metrics of repertoire skewing: heavy chain V gene (VH) usage and third complementarity-determining region (CDR3) length distribution. In §§7 and 8, we turn to diversification within individual clones and discuss some of the computational approaches and challenges when analysing clonal lineages for evidence of selection. We leave some of the details of clonal lineage analysis to the separate article in this theme issue by Steve Kleinstein [1].

Although many clones are unique, one of the most surprising findings in the literature is that certain kinds of antibodies recur—they appear to be independently generated. In §9, we review some examples of recurrent expanded B cell clones and stereotyped receptors. We comment on why such clones may be enriched in the repertoires of individuals with autoimmunity and other chronic conditions, as well as why multireactive clones may serve beneficial functions in healthy individuals. In §10, we discuss some ideas regarding clonal evolution and speculate that many expanded and persistent clones are multireactive in some of their members/progeny, at one or more times during their lifespans.

## 2. Definitions of clones that focus on different stages of B cell development

**Figure 1.**
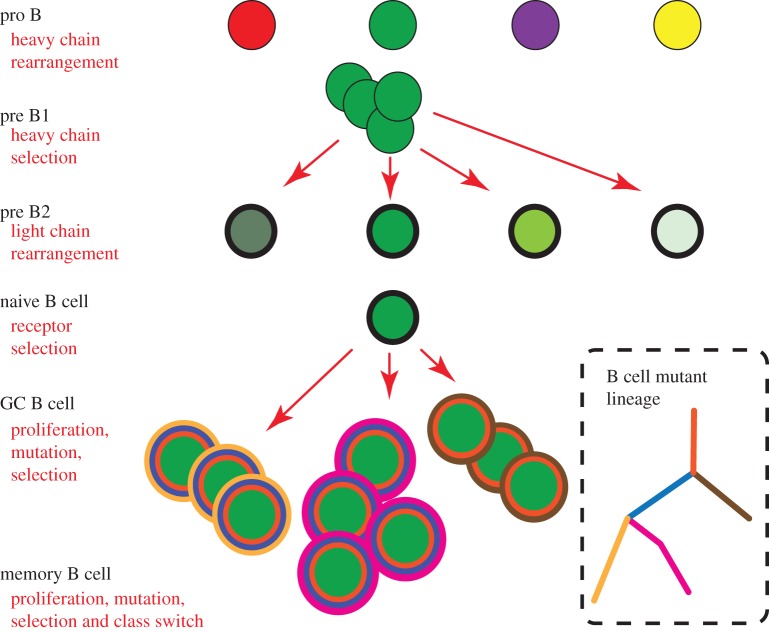
Clonal definitions at different stages of B cell development. Different stages of B cell differentiation are marked by different types of genetic recombination of antibody genes. These recombination events are retained in the genomic DNA of the B cell and can be used as markers of common ancestry for clonally related B cells (see text). At the pro-B cell stage, antibody heavy (H) chain gene rearrangement occurs. Different coloured circles correspond to B cells with different H chain rearrangements. At the pro-B to pre-B1 transition, H chains are selected. For ease of illustration, we show only a single kind of H chain (indicated by a green circle) that is favourable and B cells with this particular rearrangement undergo multiple rounds of cell division in the pre-B1 stage. In the pre-B2 stage, light (L) chain rearrangement occurs, indicated by different shades of green. Fully formed antibodies are subjected to further selection in the naive B cell stage. In the periphery, antigen-reactive B cells can undergo activation, proliferation and somatic mutation (GC, germinal centre). Somatic mutations are indicated by different coloured circles. Mutations at different branch levels are marked by different coloured circles around each ‘cell’. An appropriate lineage is constructed based on the number of common mutations in each of the expressed mutant types, leading, in this particular example, to the suggestion of a lineage with a common trunk (orange inner circle), two branch levels, and two inferred nodes (intermediary mutants).

During somatic B cell development, there are several developmental stages where clones could be separated from other clones. An overview of B cell maturation is provided in figure 1 and is based mostly upon mechanistic studies in the mouse and in cell lines [2,3]. Figure 1 summarizes the corresponding antibody gene rearrangements and other forms of diversification. Heavy chain (H chain) gene rearrangement occurs in the pro-B cell stage [4]. Developing B cells with different H chain gene rearrangements are represented by different colours. In the large cycling pre-B cell stage (pre-B1), cells with favourable H chains undergo more rounds of cell division than cells with less favourable H chains [5,6]. Favourable H chains could be those that are expressed at higher levels and/or pair more effectively with the surrogate light chain and/or signal better [5]. B cells that fail to make a productive H chain rearrangement altogether die [7]. For simplicity, we show only the fate of B cells that express a particular H chain rearrangement (denoted by dark green shading) that have successfully emerged following the pre-B1 selection checkpoint. After signalling through the pre-B cell receptor (pre-BCR) and a few rounds of cell division, these B cells are ready to enter the small resting pre-B2 stage of development, in which light chain (L chain) gene rearrangement occurs [8]. Because there is more than one B cell with the same H chain rearrangement entering the pre-B2 stage, we presume that these cells can in principle undergo independent rearrangement to different L chains and have indicated the range of different L chains with different shades of green at the pre-B2 cell stage. The next stage of B cell differentiation is the naive B cell stage, sometimes also referred to as the transitional B cell stage. These cells are the first to display the B cell receptor on the cell surface, in the form of an IgM molecule. Based largely on data in inbred strains of mice, transitional B cells appear to be short-lived and most die [9–11]. For illustrative purposes, we have therefore contracted our repertoire from many different H + L pairs in the pre-B2 stage to a single cell in the transitional stage (that has already survived the selection checkpoint within the transitional cell pool). For many surviving peripheral B cells, the next major round of selection occurs mainly in the germinal centre, where B cells are exposed to antigen and T cell help, and undergo multiple rounds of proliferation, SHM and selection for high-affinity binders [12]. Sequence variants of the clone emerge and either expand or contract, based upon their affinity for antigen(s) [13–15].

Based upon this simplified overview of B cell maturation, one realizes that there are several checkpoints at which expanded clones could be molecularly defined. If one focuses on the pro-B to pre-B cell transition, clones can be identified on the basis of having shared functional H chain rearrangements (VDJ+), as well as shared non-functional rearrangements (VDJ−) or partial rearrangements (DJ). If instead, one focuses on the pre-B2 to transitional B cell checkpoint, one could use not only the H chain rearrangement(s), but the L chain rearrangements as well. L chain rearrangements can include V–J rearrangement at the kappa and lambda loci as well as rearrangements to the non-coding recombining sequence (RS) on the kappa locus. Finally, if one focuses on the mature B cell repertoire, one could not only use the combinatorial and junctional diversity of the H and L chain gene rearrangements generated in the preceding stages, but also the diversity that arises due to SHM.

## 3. Different experimental approaches for antibody repertoire analysis

Table 1 shows four different experimental approaches that are used to analyse B cell clones. There are of course other approaches, such as Southern blotting of bulk repertoires [16,17], V gene arrays [18] and mass spectrometry of secreted antibodies [19], but we chose these four methods because they are the most frequently used.

**Table 1.** Repertoire experiment types. Shown are four different types of repertoire experiments. Each experiment is classified on the basis of the type of information it provides on the antibody repertoire starting with whether full (heavy + light chains) or single chains (heavy or light, unlinked) are studied. The information generated by the different approaches is classified into that which is readily available (yes), not available in all instances or only with difficulty (partial) or unavailable (no), see text. H, antibody heavy chain; L, antibody light chain; Ab, antibody.

method	hybridoma (H + L)	phage display (H + L or unlinked)	single cell (H + L)	bulk sequencing (H or L)
features				
all Ab loci in single cell	yes	no	partial	no
expression of Ab	yes	yes	yes	no
sort for sub-population	partial	yes	yes	yes
select for antigen-binding	yes	yes	partial	partial
study many clones	no	partial	partial	yes

### (a) Hybridoma panels

**Figure 2.**
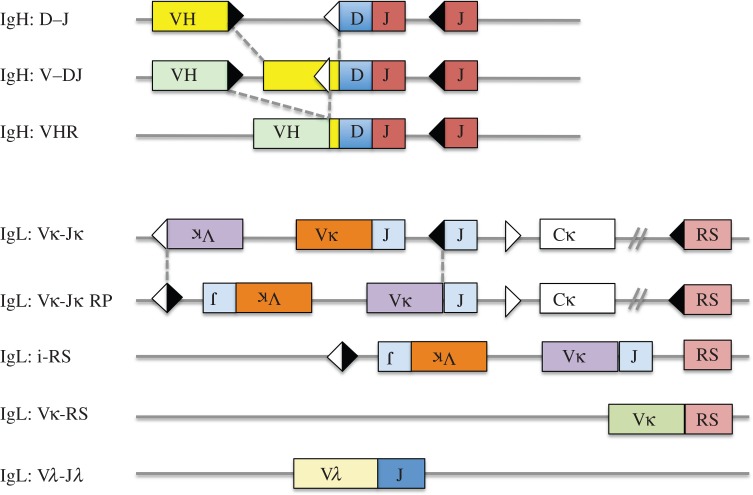
Schematic of antibody H and L chain gene rearrangements. The full range of rearrangement products that can be generated at the H chain and L chain loci is shown. In the case of the heavy chain locus (IgH), there are three kinds of rearrangements: D to J rearrangements, V to DJ rearrangements and VH replacements (VHR). Note that all H chain rearrangements are deletional and that once a complete VDJ rearrangement has taken place, all of the D gene segments are consumed. During VH replacement, an upstream VH gene can invade into a pre-existing VDJ rearrangement via a cryptic heptamer (white triangle) that is located in the 3′ end of most VH genes. VH replacement has the potential to elongate the CDR3, because the 3′ end of the preceding VH gene is usually retained in the rearrangement. For the L chain loci (IgL), the kappa locus can undergo primary Vκ–Jκ rearrangement, leapfrogging rearrangement or recombining sequence (RS) deletional rearrangement. In the case of the leapfrogging rearrangement shown, rearrangement of an upstream Vκ gene to a downstream Jκ gene occurred by inversion. Inversional rearrangement retains the original Vκ–Jκ rearrangement on the chromosome in an inverted orientation. This remnant rearrangement is referred to as a reciprocal product. The κ locus can also undergo deletion by rearrangement to RS, a non-coding sequence that is approximately 25 kb downstream of Cκ. RS rearrangement can occur via the cryptic heptamer in the JC intron (i-RS) or by deletional rearrangement of a Vκ gene to RS. Both types of rearrangements inactivate the κ locus by deletion of the constant region exon, Cκ. Finally, lambda (λ) L chain rearrangement can occur. Most λ-expressing B cells have undergone RS deletion on one or both κ alleles. All of these rearrangements can be tracked and used to evaluate clonality, particularly in hybridoma studies (see text). Squares indicate exons, triangles recombination signal sequences, fused triangles represent signal joins and fused boxes indicate coding joins. Dashed lines indicate regions where recombining gene segments come together.

Hybridomas are non-secretory myeloma cells fused to normal B cells [20]. They provide virtually unlimited amounts of genetic material, and because they secrete the antibody of the normal B cell, they provide a facile means of studying that antibody. Because of the abundance of available genetic material, the gene rearrangements at all of the antibody loci can be studied in hybridomas. The variety of antibody gene rearrangements that can be used to identify and track expanded B cell clones are shown in figure 2. To define clonally related hybridomas, one can readily generate full-length sequences of the expressed H and L chains. The analysis of these sequences can yield information about the hypervariable third complementarity-determining region (CDR3), the H + L chain pair, and even about SHMs within the variable region genes of the H and L chains. The sizes of the DNA fragments that contain various gene rearrangements by Southern blotting can be used as another clone-specific genetic feature of hybridomas. One can also perform polymerase chain reactions (PCRs) to genotype the expressed H and L chain rearrangements, and the non-productive or deletional rearrangements (such as non-productive VDJ rearrangements on the non-expressed H chain allele or RS rearrangements at the kappa locus). One can also characterize reciprocal products, such as Vκ–Jκ rearrangements that are retained on the chromosome when a subsequent inversional rearrangement occurs between an upstream Vκ and a downstream Jκ gene segment. Although nearly all of these other rearrangement products are not as diverse as the H chain CDR3, finding concordance among them in two different hybridomas is a powerful indication that the hybridomas are clonally related [21].

The major drawback of using hybridomas for antibody repertoire analysis is that they only represent a very small portion of the total B cell repertoire. There are several reasons for this. First, some kinds of B cells are easier to fuse than others. For example, in some cases, B cell mitogens such as lipopolysaccharide (LPS) are used to enhance the recovery of hybridomas and some cells, such as marginal zone cells, are more responsive to LPS than others [22]. Second, the yield of generating hybridomas in our experience is at best several hundred per mouse. Because only one in several thousand B cells successfully fuses with the myeloma cell and is propagated in culture, there is limited sampling of the repertoire. Third, hybridomas are typically selected by binding to antigen. On the one hand, this is an advantage, because multiple rounds of selection can be used to not only select for binding to a particular antigen but also to select against binding to other antigens. But a disadvantage of screening clones for the antigens they bind is that one may inadvertently discard members of the same clone that have accumulated somatic mutations that have caused their specificity to drift. Discarding clones or sequence variants within clones that bind to antigens other than the antigen of interest may result in the selective loss of B cells that are multireactive. Another difficulty is that selection for antigen binders further reduces the number of clones from several hundred to at best a few dozen per mouse. Yet even at this level of sampling, it is remarkable how many fundamental insights into clonal selection have been gained from hybridoma analysis [23–25].

### (b) Phage display

DNA that encodes antibodies (typically the single chain variable fragment) can be inserted into the genome of the bacteriophage (a virus that infects bacteria) and expressed on the surface of the virion [26]. Phage particles that express antibodies that bind to the antigen of interest are selected by panning and then propagated in bacteria. Multiple rounds of panning can be used to recover bacteriophages that harbour antibodies with the specificities of interest. Thus, phage display is a powerful tool for recovering antibodies that bind to specific antigens.

Unlike hybridomas, antibody phage display is easily adapted to recover antibodies that have different H chain isotypes and sorted subsets of B cells can be used to generate phage display antibody libraries. However, current methods have not yet resulted in the engineering of phage in which the H and L chains of the antibodies produced by single B cells are associated. Thus, phage display may generate a broader repertoire of antibodies (with random H + L pairs) than the true antibody repertoire (in which the association of specific H + L pairs can occur).

### (c) Single cell cloning

In single cell cloning, suspensions of single cells of interest (e.g. influenza-specific plasmablasts, [27]) are sorted into individual wells, RNA is extracted, and antibody H and L chain V genes are amplified. The amplified V genes are then cloned into expression vectors, transfected into cell lines and secreted antibodies are purified and analysed (further details of the protocol can be found in Tiller *et al*. [28]). Drawbacks of the method are that it is low throughput and arduous to go from H + L sequences to cloning antibodies, expressing them and testing their specificities. However, recently, some groups have developed methods for moderate- to high-throughput sequencing of H + L chain pairs from the same cell, which may substantially improve future repertoire studies with this method [29,30].

### (d) Bulk sequencing of H chains or L chains

Several recent studies of the antibody repertoire have relied upon high-throughput sequencing of antibodies from populations of B cells [31–35]. The major advantage of this approach is that very large numbers of clones can be studied and large numbers of variant sequences can be evaluated within expanded clones. When coupled with flow cytometry, this technique can also be used to evaluate B cell repertoires in different B cell subsets [36] or in B cells that bind to a particular antigen [37]. The potential to study both antigen-selected and unselected cells from the same individual and survey up to millions of different antibody gene rearrangements provides insights into the repertoire in an unprecedented level of detail.

Unlike hybridomas and antibody phage display, antigenic selection of B cells analysed by bulk sequencing is limited to a single round of selection because once the cells are selected, they are destroyed in the process of nucleic acid extraction. If antibody H chains or L chains are sequenced separately, it is impossible to recreate the H + L pairs that are associated with single cells, although bioinformatic approaches are being used to try to match H chains with L chains based upon their frequencies and other properties [38]. Technical developments using emulsion PCR-based approaches now bring moderate-throughput next-generation sequencing of full antibody (H + L chain pairs) from single cells within reach [39].

## 4. Identification of clones in high-throughput sequencing data

Most single chain bulk sequencing experiments focus on the antibody H chain. H chains are more diverse than L chains, providing a more reliable signature for clonal relatedness. H chains are more diverse than L chains because they have two rearrangement junctions in the CDR3 and these junctions are more diverse because the enzyme terminal deoxynucleotidyl transferase, which creates N additions, is more active during H chain rearrangement [40]. The H chain CDR3 also includes the D gene segment (which L chains lack). D genes can be read in up to six different reading frames and can occasionally undergo D–D fusion [41]. Finally, H chains also may undergo higher rates of SHM than L chains, particularly if there is concomitant peripheral L chain editing [42]. Higher mutation frequencies in H chains can make it easier to establish clonal association of sequences based upon shared mutations using H chain, rather than L chain sequences.

After the identification of primer sequences that indicate which sample each read corresponds to, sequences are subjected to quality control. One can use software to trim the ends of the read based upon the Fastq score, and/or introduce Ns into sequences that have likely errors (low-quality scores) in the middle [43]. Next, reads are subjected to V, D and J identification using various tools such as the ImMunoGeneTics high V-QUEST server, Ig-BLAST or others [44–48]. At this point, sequences that are identical (or sufficiently similar, based upon the frequency of sequencing errors, to be considered identical) are grouped together into ‘unique sequences’. Each unique sequence has an associated number of copies or times that it has occurred. In samples with large clonal expansions (for example in B cell malignancies), the top copy number clone will be many times more frequent than the next most frequent clone. Conversely, with highly diverse repertoires, such as naive IgM + B cells in the peripheral blood [37], clones will tend to have more uniform copy number distributions and replicate sequencing of the same DNA sample will yield minimal clonal overlap. With unmutated lymphocyte populations, the number of unique sequences approximates the number of clonotypes. But with B cells that have undergone SHM, this assumption is no longer valid. Expanded B cell clones with mutations may include sequence variants that differ by several mutations from one another. Accurate clonal assignment is critical for understanding inter- and intra-clonal repertoire diversity and selection and involves complex data analysis.

A common definition of clonal relatedness is to consider all sequences with the same germline V and J identification and some similarity in the CDR3 regions as being clonally related. One of the challenges with using the CDR3 sequence for this purpose is that the germline CDR3 sequence is unknown. Not only are the junctional sequences between V and D and between D and J generated somatically, but the D gene itself is often extensively nibbled during the recombination process, making identification of all but the longest D gene segments unreliable [49]. Current methods have 50% or lower D identification rates at 10% somatic mutation frequencies [50], which is the equivalent of approximately two amino acid positions not being identical to the germline at an average CDR3 length of 14 amino acids. For these reasons, features of the CDR3 sequence, such as its length and sequence similarity, are used as indicators of clonal relatedness. CDR3 length is typically identical for clonally related sequences, although sequencing errors and naturally occurring mutations including insertions and deletions [51–53] can cause clonally related sequences of differing lengths to be misclassified as separate clones. If VH identity is used as a criterion for clonal assignment, sequences with ambiguous VH assignments can be misclassified. VH assignment ambiguities (so-called V ties) are more likely for certain VHs, such as the VH 4 family, and occur more frequently with shorter read lengths. Another challenge with any method of clonal assignment that uses nucleotide or amino acid similarity in the CDR3 or VH or both, is that different thresholds may be required when the CDR3 length is very short and/or there is a high degree of somatic mutation in the rearrangements under study. Shorter CDR3 sequences can arise more easily by chance, leading to erroneous inclusion of multiple clones into a single clone with a short CDR3 sequence.

One further challenge when comparing many sequences to each other is deciding which sequence should be compared first. All clonal identification methods that rely upon one or more metrics of sequence similarity, such as percent sequence similarity, Hamming distances or shared somatic mutations, suffer from this problem. For example, if one randomly starts to identify a clone with the first encountered unique sequence, the ‘edges' of the clone may wind up in a different place than if one uses the highest copy number sequence within the clone, which could be closer to the centre of the clone. An alternative approach is to take all the potential members of a clone, i.e. all the sequences with the same germline V gene, J gene and CDR3 length, and to create a lineage of their common mutations. While in this instance we also consider some comparisons to be ‘first’, i.e. we are comparing all the sequences in a clone to the putative germline root of the lineage, this decision is based on a specific set of assumptions and data. Under these assumptions of clonal relatedness, all those sequences that share some threshold level of mutation are considered to be members of the same clone. For example, four or fewer mutations from the germline were empirically shown to be a level of shared mutation that was possible to achieve by chance [38,49]. However, it is important to note that for different types of immune responses the threshold level of mutation that indicates common clonality may need to be changed (i.e. a higher threshold may need to be used in highly mutated repertoires, for instance in those found in people chronically infected with HIV [54]).

**Figure 3.**
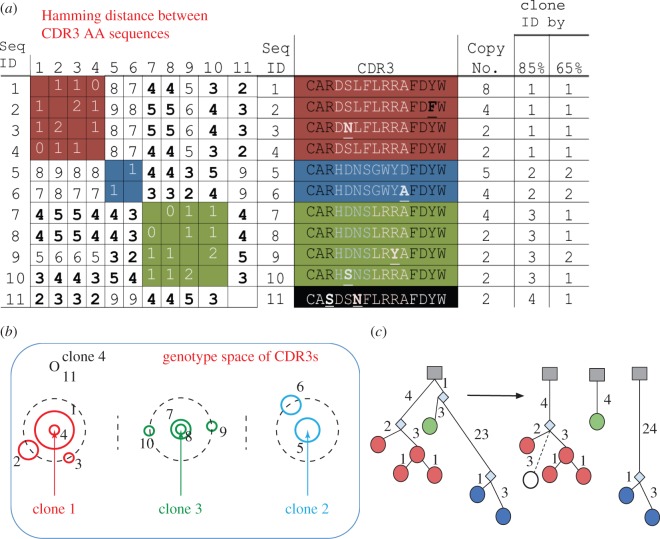
Illustration of the different outcomes for clonal identification under different thresholds of CDR3 and VH sequence identity. (*a*) CDR3s and their amino acid (AA) characteristics: the CDR3 amino acid (AA) sequence-to-sequence Hamming distances, their CDR3 AA sequences, copy numbers and clonal association using 65% versus 85% sequence identity in the CDR3, starting with the highest copy number sequence. (*b*) Clones in the genotype space of CDR3s: sequences are represented as numbered nodes. Node size corresponds to the copy number and colour reflects 85% CDR3 identity. The position of the consensus CDR3 of clones 1–3 is marked with an arrow. Space is to scale for the mutations between each sequence as in (*a*). Single and double mutations away from the consensus CDR3 positions of clones 1–3 are marked with dashed lines. (*c*) Lineage of all sequences with the same germline V and J and CDR3 length: on the left, we see the lineage of all the sequences from a single putative germline precursor (same V, and J and CDR3 length; CDR3 positions are ignored in generating the lineage as we have no consensus for these positions). On the right, we see the clonal division when the threshold of a minimal number of common mutations is set to 4. In both cases, nodes are colour coded as in (*a*). Note: each node may contain multiple sequences if they share all the mutations in V. Thus, clone 3 (green) sequences appear to share all mutations and do not diverge in this representation as they do in (*a*).

In figure 3, we show how different thresholds and methods suggested above can lead to differences in clone assignments. We consider 11 sequences that we cluster by their CDR3s at 85% identity into four clones (figure 3*a*—clone 1, red; clone 2, blue; clone 3, green and clone 4, black**)**. In every clone, all of the sequences are 85% similar in the CDR3 to all of the others (i.e. they differ from all other members of the clone by up to two mutations in CDR3; figure 3*b*). If we consistently assign sequences to clones starting with the largest copy number sequence, we get a reproducible order of clones, which we can compare with the sets of clones formed from these 11 sequences by other means. Next, we consider the same sequences under less stringent demands of CDR3 identity (65%) and this leads to a different division of the sequences into two clones (figure 3*a,b*). Alternatively, we consider all the mutations from the germline and use them to create a lineage. This method has the advantage that it considers the entire length of the sequence to assess similarity [49]. However, it cannot consider the CDR3 regions (as we do not know to distinguish germline positions in the CDR3). In this example, the lineages suggest three clones that match most of the clones from the 85% CDR3 method (figure 3*c*). Thus, we can see that decisions on which part of the sequence data to use, and how the threshold of similarity is set, change the clonal assignment of sequences. However, even with different methods, the identity of the big clones remains quite constant (figure 3). One way to potentially overcome sequence comparison ordering issues in clone identification is to empirically perform clone identification using different seed sequences, different cut-offs for similarity and/or different or iterative clustering approaches to determine if robust clonal lineages exist [38,55].

## 5. Evaluation of the clonal landscape: clone identification and tracking

Once the sequences have been collapsed into clones, the next step is to study the frequency distribution of clones in the repertoire. For example, histogram plots can be created to show the copy numbers of all of the clones in the repertoire. The copy number refers to the number of times the sequences that comprise the clone appear in the sequencing dataset. In a very diverse repertoire, as occurs in the naive B cell pool within the peripheral blood of healthy humans, the copy number distribution is fairly flat, with the highest copy number clones being minimally more frequent than the next most frequent clone. In contrast, if the repertoire contains one or more very large expanded clones, as occurs in the setting of some autoimmune diseases, such as Sjogren's syndrome [56] or systemic lupus erythematosus (SLE) following rituximab therapy [57], or B cell malignancies such as chronic lymphocytic leukaemia (CLL) [58], there will be a few or even just one highly dominant high copy number clone that is many times more frequent than the next most frequent clone.

Because clonal expansion is a fundamental feature of an immune response, we are very interested in learning whether expanded clones can be identified and tracked over time in individuals with immunologic disorders such as SLE. To begin to address these questions, we need to have a reliable means of quantifying the clone copy numbers, so that different sequencing libraries can be compared with each other. Some have approached this problem by using calibrators that are amplified along with the endogenous rearrangements. The ratio of the copy numbers from the calibrators versus the cells in the sample provides a means of normalizing the copy number [59]. But in the case of IgH rearrangements, such normalization procedures may be insufficient because SHM can affect the efficiency of PCR amplification and not every single somatic variant can realistically be captured in the calibrators. Alternatively, one could start from RNA and use primers that are in the constant region to circumvent mutation. But with RNA from bulk populations, one has to correct for differences in transcript abundance in different cell types or perform amplifications on sorted or single cells. In addition to using calibrators or some other normalization technique, one can generate replicate sequencing datasets from the same sample [60]. By performing multiple replicate amplifications from independent DNA aliquots of the same sample, one can determine the maximal expected clonal overlap within a given sample, determine which clones are reliably identified in the replicates (including the reproducibility of their copy number rankings) and, with adequate sampling, perform a rarefaction analysis to empirically determine which clones can be identified reliably [61]. A final method to assess the relative extent of clonal expansion is to count the number of unique sequences in a given clone, counting every variant of mutant sequence in a clone only once. While this is not a direct measurement of all expansion as it depends on mutation, it is nonetheless quite reliable, when mutations are present.

## 6. Metrics of repertoire skewing

In addition to the relative or absolute quantification of top copy number clones, one can also study the repertoire as a whole. Global metrics of the repertoire landscape include evaluations of how ‘skewed’ the repertoire is. One relatively simple and commonly employed metric is VH gene usage. VH usage can be quantified by calculating the percentage of clones that use a particular VH. One can also evaluate VH usage on the basis of clone copy numbers. A comparison of the VH usage of clonotypes (where each clone is only counted once) with VH usage with total copies provides insights into the effects of high copy number clones on the repertoire. VH usage is frequently shown as a heat map, and various types of clustering algorithms can be used to compare VH usage in different sequencing libraries, similar to what investigators use for microarray data.

Another general metric of repertoire skewing is the size distribution of antibody CDR3 lengths. In a diverse repertoire, we expect the distribution of the CDR3 sizes to resemble a truncated and discretized Gaussian distribution [62]. If the size distribution of CDR3s in the repertoire exhibits kurtosis or skewing, this can be an indication of an unexpected over-abundance of clones undergoing abnormal development or perhaps reacting to a specific antigen. For instance, antibody sequences with short CDR3 lengths may derive from B1 B cells, which develop in fetal life and tend to have fewer N additions because the enzyme that creates N additions (terminal deoxynucleotidyl transferase) is less active during fetal life [40]. Conversely, human antibody sequences with long CDR3 lengths may have an increased frequency of JH6 usage (JH6 has a series of tyrosine residues at the 5′ end that contribute towards the CDR3 length, as was observed in early stage B-cell precursors [63]). Another potential molecular mechanism for CDR3 elongation is VH replacement. In VH replacement, an upstream VH gene invades into a pre-existing VDJ rearrangement on the same allele [64,65]. VH replacement can elongate the CDR3, because the invasion occurs into a cryptic heptamer sequence that is upstream of the V–J junction. Thus, in some cases, the 3′ end of the VH gene in the replaced allele not only contains the original V–J junction, but also a few nucleotides from the 3′ end of the VH gene itself, the so-called VH replacement ‘footprint’ [66].

## 7. Distinguishing somatic mutation from sequencing errors

Another general metric of the repertoire is to evaluate the level of SHM, which can provide insights into the general composition of the repertoire. If a high fraction of clones has mutations, this indicates that many of the cells that were sequenced were memory cells and/or may have entered into a germinal centre reaction and received T cell help. The quantification of somatic mutations seems simple, but for this method to be robust, one has to control for technical errors that produce mutations.

A significant challenge with bulk high-throughput sequencing approaches is that sequencing has a significant error rate. How do we distinguish somatic mutations from sequencing errors and other mistakes? Most methods for doing this with DNA-based sequencing protocols involve running samples in replicates and looking for the presence of the same sequence variants in more than one replicate. Within the same sample, one can also institute a copy number cut-off. Sequencing errors are less likely to be found in higher copy number sequences, because the same error has to recur. However, with high-depth sequencing experiments, it is surprisingly easy to regenerate the same sequencing errors. Therefore, stringent filtering of high-throughput data is important for reducing spurious mutations. Fortunately, certain types of errors are more common and can be filtered from reads using computational approaches, some of which are sequencing platform-specific [43,67–73]. Because true somatic mutations are often shared among clonally related sequences, this is another approach that can be used to distinguish real mutations from sequencing errors.

One of the most rigorous experimental ways of addressing sequencing errors is to employ a technique known as molecular barcoding [74]. Barcoding involves using primers that contain stretches of random nucleotides to label amplifications of single molecules. Mutations that are shared in the majority of sequences with the same barcode are likely to be real, whereas mutations found in single sequences are likely to be due to sequencing error. Another promising approach to obtaining high-quality reads is a single molecule circular amplification technique. This moderate-throughput method generates very high fidelity sequences at very long reads up to several thousand nucleotides in length and recently was used to demonstrate novel splicing isoforms in human antibody heavy chain transcripts [75].

## 8. Evaluation of inter- and intra-clonal diversity: where are the boundaries of a clone?

**Figure 4.**
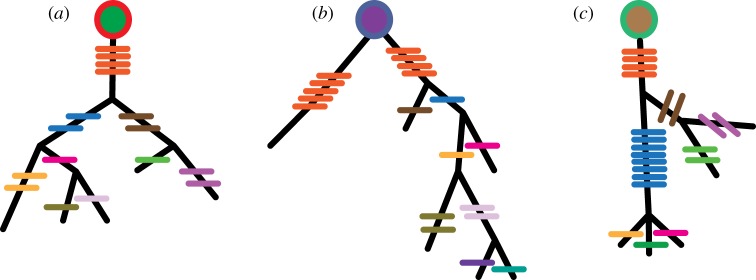
When is a clone not a clone? Illustration of three clones, each starting from a different heavy and light chain combination, as marked by the circles at the root of each lineage. Members of each clone exhibit different numbers of shared and unique mutations, marked by small coloured lines at each branch. Colours indicate how close to the root mutations are. Orange, mutations from root; blue or brown one level up from root; other colours represent leaves.

Intra-clonal diversity is generated by and large through SHM. The first step of mutation analysis is the identification of the closest germline source. It is important to identify which sets of sequences and mutations come from the same clonal source, otherwise when studying the mutations in a repertoire we will be mixing both independent and dependent events. For example, if a mutation occurs in five different sequences in a single clone, we can deduce that they are siblings that shared a single mutation in an ancestor, whereas if the same mutations had occurred in five different clones, we would count it as a mutation that appears multiple times in the repertoire. The analysis of unique mutations per clone has been used to detect selection pressure [23,76,77]. The unique mutations in a clone can be considered together to create a lineage of mutations that describes the process of clonal development (figure 4). Analysing the patterns of these lineages can give insights into the diversification and selection processes that lead to clonal evolution [78,79]. Different lineage analyses have led to the calculation of the mutation rate of SHM [80] and as metrics of the specific selection pressure on a set of mutations in a given branch or at the root of the lineage [81,82].

One issue in creating and analysing clonal lineages is that we cannot be certain of our identification of mutations. Errors in identification can stem from experimental error, but also because of inherent real limitations of our knowledge. While the recombined heavy and light chains are diverse, V genes, D genes and J genes are quite similar and can generate mutants that are closer to germline genes that are not their true origin. In principle, as germline identification is error-prone, it may be better to identify sets of clonally related genes by showing that they minimize some function of co-similarity rather than aligning every sequence first to the germline and using that characteristic to identify clones. While we cannot do this across all sequences, it may be feasible to do so to verify existing indications of clonality (much as we describe above in our lineage method for clonal identification). In this instance, the comparison with a germline gene is not used just to identify mutated positions in every sequence, but rather is also treated as a context under which we check if the differences between sequences in the clone are minimized [83]. Thus, we can estimate the efficacy of different clonal identifications in an analysed repertoire and potentially identify instances when clones are not well defined and should be further analysed or compared. A recently described algorithm for constructing lineage trees, called ImmuniTree, explicitly models SHMs, sequencing errors and iteratively evaluates internal nodes of the tree in an effort to generate robust clonal lineages [38].

There has been a heightened interest in using metrics from ecology to study the inter-clonal diversity of clones in immune repertoires [84,85]. However, at present such experiments can only reliably evaluate highly expanded clones, for instance by focusing only on the Ig G subset of the response to the hapten (4-hydroxy-3-nitrophenyl)acetyl [60]. Diversity is most commonly measured using indices such as the Shannon entropy or by using the ‘true’ diversity, which, when counted so as not to be biased by small or large clones, is equivalent to the exponential of the Shannon entropy [60,86]. There are also various statistical methods to ascertain the minimal clone size (in terms of sequence number or fraction of the sequenced repertoire) at which diversity can be estimated reliably. These include estimates of re-sampling likelihood (per clone of a given size, [87]). When sampling an environment (or the antibody repertoire) one can construct an accumulation curve of the species (or clones) identified. This curve is either based on the tracking of individual sequences, when considering a single sample where each individual sequence is acquired separately, or by sample if we consider multiple sampling experiments in which a subset of individual sequences are taken at once. In both cases, we can calculate the estimated or *rarefaction* curve of the order free sampling of the environment in our samples [61,88,89]. If the rarefaction curve plateaus, we can reliably estimate the diversity. Rarefaction is a better and more computationally efficient method for estimating if sampling is sufficient than performing random re-sampling by simulation [87,90], as these latter methods are simply a numerical approximation of the estimate that rarefaction calculates directly.

## 9. When is a clone really more than one clone?

As the number of independent sequences that are sampled increases, the chances of finding similar sequences that may arise independently increases. Similar to the parlour game where one is asked to estimate the probability of any two people in the room sharing a birthday, we can determine the probability of any two clones sharing a particular H chain rearrangement by chance. To make this calculation, we need to estimate how many different (heavy chain) CDR3 sequences can be generated. If we assume that the whole CDR3 is determined by 49 V, 27 D and 6 J genes alone, that the frequencies of V/D/J gene usage are uniformly distributed, that the same outcome cannot be achieved through multiple combinations of different Vs, Ds or Js, and that D segments can be read in six reading frames (three forward and three reverse), then the probability of having the same heavy chain is 1/49*1/6*1/(27*6). In a single experiment with 10 000 sequences, this translates to an approximately 20% probability of finding at least one instance of the same CDR3 twice by chance. However, the addition of non-templated nucleotides and exonucleolytic nibbling at the junctions between the recombining gene segments makes the probability much smaller. If there is even one amino acid not accounted for by the germline genes, the probability of encountering two different clones with the same CDR3 is reduced to approximately 1% and with two amino acids, it is further reduced to approximately 5 in 10 000. This is probably still an overestimate of how many independently generated similar clones we will find.

Statistical estimates of CDR3 sharing have been described for T cell receptor (TCR) sequencing data [91–93]. However, it is difficult to extrapolate from T cell repertoire diversity to B cell repertoire diversity because of differences in rearrangement (such as the frequency of D–D fusion events, which occur in approx. 2% of productive TCRβ rearrangements [94] but in only approx. 1/800 IgH rearrangements [95]), potential differences in the extent of clonal expansion, and differences in that only B cells undergo SHM. Estimates of BCR diversity have been made indirectly using phage display to provide high-quality DNA libraries for deep sequencing and reveal that not only the hypervariable CDR3 sequence but also somatic mutations in CDR1 and CDR2 of the V gene contribute substantially to the overall BCR repertoire diversity, which was estimated to be at least 3.5 × 10^10^ different clonotypes [96]. More recently, the frequency of shared CDR3 sequences in memory B cells from different individuals was observed to occur at a frequency of approximately one in 4000 clonotypes [74]. Most of these recurrent instances of clones were likely the result of rare recurrent recombination and not selection as they were mostly un-switched, un-mutated and had short CDR3s [74].

These estimates appear to indicate that occurrences of independently generated overlapping CDR3 sequences are quite rare, although if we consider multiple samples from multiple experiments, the number will increase. However, it is important to note two caveats to this low estimate: (i) these calculations assume full knowledge of the source of the CDR3 positions. In reality, owing to sequencing errors and the difficulty in identifying D gene associations [49], we must be satisfied with identifying all sequences that have a CDR3 that is ‘close enough’. (ii) To make our calculation, we assume that all combinations are equally likely to occur (that they are uniformly distributed), but this is not true. For example, some VH genes (such as the 3′ VHs in fetal life, [97]) rearrange more frequently than others. Moreover, repertoires from mature or memory B cells have undergone additional rounds of selection; IgH rearrangements that are positively selected will be more numerous owing to clonal expansion.

The erroneous classification of two independent clones as a single clone can also occur. The generation of clonally distinct yet highly similar antibodies may be more likely under certain circumstances. B1 B cells tend to harbour shorter CDR3 sequences, exhibit skewed VH gene usage and at least a subset of B1 cells can have low levels of SHM [98,99]. All of these properties make it easier for B1 B cells to harbour highly similar antibodies than B2 B cells. A second circumstance could arise if different L chains rearrange in different clones that actually share the same antibody H chain. This hypothetical scenario is possible because pre-B1 cells undergo multiple rounds of cell division prior to the initiation of L chain rearrangement [5]. In a clone with a favourable H chain, there could be as many as seven rounds of rearrangement, generating up to 128 pre-B cells with the same H chain. If L chain rearrangements in B cells with the same H chain gene rearrangement occur independently, then the same H chain could be found in combination with many different L chains. Consistent with this idea, studies with the E12 cell line, which can differentiate from the pro-B stage all the way to B cells in culture, demonstrate that subclones with the same VH rearrangement can have different L chain rearrangements [100]. An interesting prediction of this theory is that, independent of the H chain specificity, the number of rounds of pre-B cell division could dictate how many different L chain partners a given H chain can have. A third setting in which similar or identical H chains could be independently created occurs when there is positive selection for independent B cell clones with shared amino acid sequence motifs that bind to one or more particular antigens. Such sharing has been given a variety of names including ‘public CDR3s’ and ‘stereotyped receptors’ and has been documented in many different chronic disorders ranging from infections and autoimmunity to cancer. A few examples are highlighted in table 2. Table 2 is not an exhaustive list of stereotyped receptors from the literature. Here, we have decided to focus on cancer and autoimmunity as these represent chronic responses to internal or modified self-antigens. However, there are other examples of sharing of antibody sequence motifs, for example in responses to repetitive polysaccharides such as the *Haemophilus influenza* type B polysaccharide [114,115] or to viruses such as influenza [27,55]. Some of these immune responses could be chronic as well and/or use antibodies that are positively selected for binding to some other (unknown) self-antigen.

**Table 2.** Shared sequence motifs in clonally unrelated antibodies from humans. Recurrent antibody specificities and/or structural features are summarized in this table and some examples (CLL) are discussed in more detail in the text. The examples are meant to be illustrative rather than exhaustive and represent only a small fraction of shared antibody motifs and heavy and/or light chain restriction that can be found in the literature. ADAMTS13, a distintegrin and metalloproteinase with a thrombospondin type 1 motif, member 13; B-NHL, B cell non-Hodgkin lymphoma; CLL, chronic lymphocytic leukaemia; FR1, framework region 1; HCV, hepatitis C virus; I/i antigens, red blood cell antigens (I has a linear chain of *N-*acetyllactosamine units); ITP, immune thrombocytopenic purpura; MALT, mucosa-associated lymphoid tissue; NAL, *N*-acetyllactosamine; LDL, low density lipoprotein; RBC, red blood cell; RF, rheumatoid factor; SLE, systemic lupus erythematosus; TRIM21, tripartite motif-containing protein 21; TTP, thrombotic thrombocytopenic purpura; Wa Id, Wa idiotype.

disease	reported specificities	H + L	comments	references
B cell malignancies				
CLL	oxidation-induced malonedialdehyde on LDL, apoptotic blebs and some microbes; non-muscle myosin H chain IIa associated with apoptotic cells; *S. pneumo;* *H. influenzae;* Gram-negative bacteria; anti-DNA, *N-*acetyl-lactosamine epitope recognized by VH4–34 Abs	VH1–69/D3–3/J6 VH3–23 VH3–21 + V*λ*3–21 VH4–34 + V*κ*2–30	mutational status as well as specific rearrangements are associated with disease prognosis; stereotypy can involve the specific VDJ, H + L chain pair and/or CDR3 sequences, or some combination of all three	[101–105]
Burkitt's lymphoma	staphylococcal protein A, I/i antigens (NAL)	VH4–34	hydrophobic patch in FR1	[106]
splenic marginal zone lymphoma		VH3–21 VH4–34 VH1–8 VH3–23		[107]
MALT lymphomas	rheumatoid factors that bind to IgG-Fc; presence of these RFs in patients with Sjogren's syndrome is associated with increased risk of B-NHL	VH1–69/JH4 (Wa Id) VH3–7/JH3 VH4–59/JH2	stereotyped RFs can also be seen in HCV patients with cryoglobulinemia or lymphoma, donors immunized with mismatched RBCs	[108]
autoimmune conditions				
Sjogren's syndrome	anti-Ro52 (TRIM21) antibody proteomes	VH3–23 or VH3–7 paired with Vk3–20		[109]
SLE	I/i blood group antigens; FR1 binds NAL; apoptotic self-Ag dsDNA, chromatin, cardiolipin	VH4–34 (9G4Id) L chain differences	hydrophobic patch in FR1 of VH4–34 L chains A3/A19, A27, O11a, 2–8, 3–2 and 5–1 are more common in SLE than controls	[18,110]
TTP	ADAMTS13	VH1–3 VH4–28 VH1–69 VH3–30 shared motifs in CDR3		[111]
ITP	anti-platelet	VH3–30	many anti-platelet antibodies bind to GPIIb/IIIa	[112]
pemphigus vulgaris	desmoglein 3	VH1–69 VH1–46	DFDHW motif in CDR3	[113]

Some of the most fascinating and well-characterized shared antibody motifs occur in CLL. Up to one-third of patients with CLL have ‘stereotyped’ antibodies [101,102]. Stereotypy can refer to sharing of the VDJ genes of the H chain V region or VJ of the L chain, or to specific H + L chain pairs and/or to specific amino acid motifs within the V, particularly but not exclusively in the CDR3 [103]. The sharing of motifs suggests that there may be common antigens that drive the disease. Consistent with this idea, three-dimensional modelling based upon publicly available structural data on H + L chain variable region pairs from over 300 CLL patients revealed a restricted series of predicted antigen-binding sites [116]. While this suggests that the repertoire of potential antigens is limited, there is also evidence to suggest that CLL B cells with stereotypic antibodies share many features with B1-like cells, including multireactivity [117]. For example, in TCL transgenic mice (an animal model for CLL) expanded B cell clones are often polyreactive and harbour specificities for phospholipids, lipoproteins and/or polysaccharides [118]. In human CLL patients with subgroup 1 antibodies, the BCRs recognize oxidized LDL, but fail to respond fully to BCR crosslinking *in vitro* [119]. Other CLL antibodies recognize antigens on apoptotic cells, similar to multi- and autoreactive B cells [104]. In fact, some VH4–34 antibodies in CLL look very similar to VH4–34 antibodies in lupus: they can have a hydrophobic patch in framework region 1 that binds to *N*-acetyllactosamine (NAL) [110,120]. Also resembling VH-4–34 antibodies in lupus, some of the VH4–34 antibodies in CLL can have positively charged residues (arginine, lysine) in the CDRs, reminiscent of anti-DNA antibodies in lupus [121]. Intriguingly, some CLL cases have more than one functional L chain rearrangement, which could be due to failed feedback inhibition of rearrangement or to active (auto)antigen-induced receptor editing [105].

Many examples of public or stereotyped receptors are multireactive. Why is this so? One possibility is that many antibodies are multireactive, once we fully characterize their specificities. Multireactive antibodies are readily created during primary B cell maturation [63,122], and are also created by clonal selection and SHM [123]. It is also conceivable that B cells that produce multireactive antibodies can rely on several different potential mechanisms for survival, some of which may be co-opted or enhanced by chronic infections, cancer or autoimmunity. For example, in the case of autoimmune disease, multireactive B cells may respond to self-antigens expressed by dead or dying cells, contribute to inflammation and cell injury, thereby creating a feedforward loop of self-antigen, immune stimulation and further self-injury [124]. By binding to complex (auto)antigens that may simultaneously engage pattern receptors such as TLR9 and/or TLR7, multireactive B cells may be able to circumvent traditional tolerance mechanisms. Furthermore, some of the antigens that these cells bind (such as nucleic acid, or other antibodies in the case of rheumatoid factors) can form complexes that can link with a much broader diversity of antigens, permitting the multireactive B cells to present a wider array of peptides that can be used to recruit (inappropriate) T cell help [125].

But all of this begs the question of why multireactive antibodies are not only easily generated, but frequently observed also in healthy individuals with properly regulated immune systems. In health, B cells with multireactive antibodies appear to have protective as well as anti-inflammatory functions [126]. A famous example of an anti-inflammatory antibody is the T15 idiotype (encoded by the murine S107/TEPC15 V1 gene), which is an IgA antibody that binds to *Pneumococcus* C polysaccharide, and was shown by immunodiffusion to bind to lipids containing phosphorylcholine [127]. B cells expressing the T15 idiotype have very limited junctional diversity and can respond in a T cell-independent manner to bacterial antigens, serving a protective role in early life [128]. But T15 also binds to self-lipids, including oxidized LDL and has amino acid motifs that resemble other self-antigens associated with inflammation including C reactive protein, which is an acute phase reactant [129]. T15 can even bind to itself (it is an ‘autobody’ [130]), which could have autoregulatory functions and/or allow B cells to undergo BCR signalling in the absence of antigen engagement. In genetically engineered mice, the T15 antibody is anti-inflammatory, promoting apoptotic clearance, reducing atherosclerosis and suppressing TLR responses [126,131,132].

In addition to forming at high frequencies in the primary repertoire, where they are sometimes termed ‘natural’ autoantibodies, multireactive antibodies can also be created and selected for by SHM. For example, the well-known 3H9 antibody binds not only to DNA, but also to cardiolipin [23] and dioleoyl phosphatidylserine [133]. Phosphatidylserine is normally confined to the inner leaflet of the cytoplasmic membrane, but becomes expressed on the cell surface in apoptotic cells, where it is recognized by phagocytic cells [134]. Increased binding to phosphatidylserine was observed in sequence variants of 3H9 that had arginine residues that had been shown previously to enhance DNA binding, suggesting that DNA binding and phospholipid binding are co-selected [133]. When dysregulated in autoimmunity, multireactive B cells may be very hard to eliminate because of the selected functions of their healthy ‘natural’ counterparts in apoptotic cell clearance, reduction of inflammation and protective immunity. Multireactive B cells may also benefit from several different means of survival, through interaction with various self- and non-self-ligands and through different activation pathways that may involve or circumvent T cell help.

## 10. Markers of clonal evolution/distinctive selection under conditions of autoimmunity

We have described herein how diversification of the genes encoding the BCR can be used for clonal definition and can be analysed to make observations about clonal development. Here, we would like to discuss the limitations of clonal thinking in terms of its impact on repertoire change and functional specificity. By segregating sequences based upon their history of diversification, it is implied that they share some function or purpose. This assertion need not be true, however. When we measure a repertoire of mutant B cells, we are querying the history of cells that have already survived selection. When thinking of a prototypical example of how a clone develops, we would expect all surviving mutants of the same clone to share a set of common mutations at their origin which would later diverge with a few individual mutations for each mutant (figure 4*a*). This divergence occurs either because not enough time has passed to select only one outcome or because, following these initial key mutations, survival is not dependent on a gain of affinity, but only on maintaining it, and so a wide range of neutral mutations can sustain it [135].

However, this is not the only possible scenario of how a clone can diverge by mutation. Other options lead to outcomes where the common function of members of the expanded clone is less clear. The first such example arises when there are two sub branches of a clone that share the same germline source but no common mutations (figure 4*b*). In most cases, we would consider this to indicate that there are actually two clones that arose independently and were mistakenly identified as being one. In our final example (figure 4*c*), we see a clone in which all members share a few origin mutations but then diverge extremely. In this instance, the divergence is exemplified by ten mutations shared by all of the sequences in the left subclone. However, the divergence could be a deletion found in only a subset of sequences in a clone, or a different pattern of mutation types in different regions of the lineage. These differences could be linked to different clonal outcomes, such as cancer or autoimmunity, in which cells either expand or persist without sufficient constraints.

**Figure 5.**
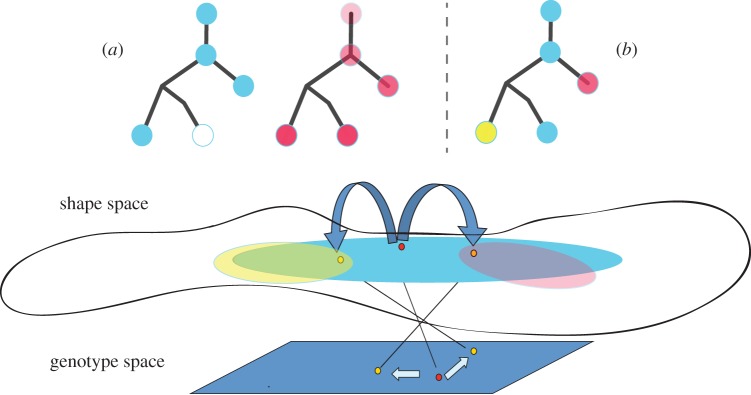
The common affinity of a clone: somatic mutation and selection can be considered as a movement on genotype space that leads to reciprocal changes in a related shape space. Thus, through mutation members of a clone traverse regions of different antigen specificity and affinity, from regions of more multireactive binding (light blue) to areas of greater specificity to particular antigens (pink or yellow). We can envision two scenarios of clonal evolution. (*a*) Clones start and continue in the same vein either improving their affinity to specific antigens (more opaque pink nodes on right) or maintaining some level of general (low) cross-reactivity (left lineage with blue nodes). (*b*) A clone that shifts it affinities and specificity over its lifetime.

While this last example is extreme, it probably exemplifies some basic characteristic of most large expanded clones in immune repertoires. As we can now observe with high-throughput sequencing, clones can be widely dispersed. Even if they share substantial mutation backgrounds, their end branches can be quite divergent. We would suggest that this is linked to a functional divergence. When we consider how clones expand and improve their affinity with time we can consider two scenarios: (i) each clone has a specific affinity or is multireactive (figure 5, yellow- and pink- versus blue-shaded lineages), and mutation and selection improve this affinity or (ii) all clones start being multireactive but have a weak affinity of interaction for one or more specific antigens, and this specific interaction is selected for over time, making the antibody more specific and potentially less multireactive.

As with theories of evolution, it may be naive to think of affinity maturation as being simply a matter of ‘survival of the fittest’. Only clones and mutants that have survived previous rounds of central and peripheral selection will participate in further rounds of affinity maturation and so we can predict that the shape space they occupy should be relatively flat, i.e. there is some baseline level of responsiveness that is robust to mutation [136]. How does this translate to antigen receptor repertoire selection? It has been suggested that to avoid negative selection, TCRs should be comprised many weak interacting amino acids that bind to strongly interacting amino acids in the antigen, creating a set of moderate strength interactions that are needed to create a strong overall avidity of binding [137]. Because even the difference of one amino acid in the antigen can degrade this type of binding, it leads to high specificity. In B cells, however, the receptor–antigen interactions are more complex, making it hard if not impossible to predict exact amino acid interactions in the BCR. Yet, in spite of this, the overarching need to avoid negative selection and maintain a repertoire that can change without mutating into an unusable form remains. Thus, although we cannot predict exactly how mutations will change the contact positions of the antibody [138], we can speculate that many successful clones that persist over time may at some point in their lifespan be multireactive.

## Data accessibility

This is a review paper and does not contain primary data.
